# Relevance of anaerobic bacteremia in adult patients: A never-ending story?

**DOI:** 10.1556/1886.2020.00009

**Published:** 2020-06-05

**Authors:** MÁRIÓ GAJDÁCS, EDIT URBÁN

**Affiliations:** 1 Department of Pharmacodynamics and Biopharmacy, Faculty of Pharmacy, University of Szeged, Eötvös utca 6., Szeged, 6720, Hungary; 2 Department of Public Health, Faculty of Medicine, University of Szeged, Dóm tér 10., Szeged, 6720, Hungary; 3 Institute for Translational Medicine, Medical School, University of Pécs, Szigeti út 12., Pécs, 7624, Hungary

**Keywords:** bacteremia, bloodstream infections, blood cultures, anaerobes, MALDI-TOF, *Bacteroides*, *Clostridium*

## Abstract

Obligate anaerobic bacteria are considered important constituents of the microbiota of humans; in addition, they are also important etiological agents in some focal or invasive infections and bacteremia with a high level of mortality. Conflicting data have accumulated over the last decades regarding the extent in which these pathogens play an intrinsic role in bloodstream infections. Clinical characteristics of anaerobic bloodstream infections do not differ from bacteremia caused by other pathogens, but due to their longer generation time and rigorous growth requirements, it usually takes longer to establish the etiological diagnosis. The introduction of matrix-assisted laser desorption-ionization time-of-flight mass spectrometry (MALDI-TOF MS) has represented a technological revolution in microbiological diagnostics, which has allowed for the fast, accurate and reliable identification of anaerobic bacteria at a low sample cost. The purpose of this review article is to summarize the currently available literature data on the prevalence of anaerobic bacteremia in adults for physicians and clinical microbiologists and to shed some light on the complexity of this topic nowadays.

## INTRODUCTION

Under physiological conditions, obligate anaerobic bacteria are considered important constituents of the microbiota of humans; on mucosal surfaces and in some anatomical regions (oral cavity, female genital tract, colon) their numbers exceed the number of facultative anaerobes by a magnitude of 10–1,000 [1, 2]. These strict anaerobes have a protective role against obligate pathogenic bacteria by consuming nutrients in the anatomical niche and by secreting short-chain fatty acids (SCFAs); this phenomenon is called colonization resistance [3]. Bacteria should be classified as strict anaerobes, if they are unable to replicate (i.e., to form colonies) on solid media in the presence of atmospheric oxygen (18% O_2_ and 10% CO_2_) [4]. The relevance of anaerobic bacteria as pathogens has been described from basically all anatomical areas, these infections may be divided into two main groups: exogenous or “classical” infections (botulism, gas gangrene, lockjaw) are predominantly monomicrobial, toxin-mediated diseases, where the principal causative agents are spore-forming Gram-positive rods (i.e., members of the *Clostridium* genus), while so-called endogenous or “modern” infections are mainly polymicrobial (mixed aerobic-anaerobic) infections, where the components of the normal bacterial microbiota are seen as pathogens [1, 2, 5, 6]. The following anaerobes are accountable for the majority (>90%) of clinical infections: Gram-negative rods *(Bacteroides/Parabacteroides* spp., *Prevotella* spp., *Porphyromonas* spp., *Fusobacterium* spp., *Bilophila* spp., and *Sutterella* spp.), Gram-positive spore-forming *(Clostridium* spp.) and non-spore-forming *(Actinomyces* spp., *Bifidobacterium* spp., *Eubacterium* spp., and *Cutibacterium (Propionibacterium)* spp.) rods and Gram-positive anaerobic cocci (GPAC) and Gram-negative cocci *(Veilonella* spp. and *Megasphera* spp.) [7–11]. Based on the source of the infection, *Bacteroides/Parabacteroides* spp. and *Clostridium* spp. mainly originate from the gastrointestinal tract, GPAC, pigmented *Prevotella* spp., *Porphyromonas* spp., and *Fusobacterium* spp. arise from the upper airways, from pulmonary sources or the female genital tract, while *Cutibacterium acnes* mainly originated from the skin and foreign bodies [1, 2, 12].

Several risk factors have been identified for the development of anaerobic infections, such as cancer (solid tumors or hematological malignancies), immunosuppression associated with organ transplantation, corticosteroids, cytotoxic agents or other types of immunosuppressing factors (e.g., splenectomy, diabetes mellitus), gynecological, gastrointestinal surgery or presence of decubitus ulcers [1, 2, 5, 6, 13]. From a clinical standpoint, anaerobic infections should be suspected if the patients present with one or more of the following: poor oral hygiene, foul-smelling discharge, suppuration, abscess formation, thrombophlebitis, tissue destruction adjacent to relevant mucosal surfaces, infectious processes related to malignant diseases (with no growth in aerobic cultivation methods), free gas in the affected tissue (characteristic for gas gangrene), and sulfur granules in histopathology (characteristic for actinomycoses) among others [1, 2, 5, 6, 13, 14]. The clinical relevance of anaerobic bacteria was further strengthened by the emergence and spread of *Clostridioides difficile* (especially the hypervirulent, 027 ribotype), which is currently considered as one of the most prevalent enteric nosocomial pathogens in the 21st century and an important factor of mortality [15, 16]. The therapy of anaerobic infections is selected on empirical basis in most cases (with *β*-lactam antibiotics, metronidazole or clindamycin being the most frequently used agents), which is possible due to the more or less predictable resistance patterns of these pathogens [17–19]. However, some species possess genetically-determined resistance mechanisms (e.g., metronidazole-resistance in the aerotolerant genera of Gram-positive rods, macrolide and rifampin-resistance in *Fusobacterium* spp., cefoxitin-resistance in *C. difficile)*, and the development of resistance against *β*-lactam antibiotics (conferred by the *cfx*A and *cfi*A genes) and metronidazole (conferred by the *nim*A-K genes) is concerning [13, 19–22]. The number of multidrug resistant (MDR) anaerobic strains, most frequently members from the *Bacteroides/Parabacteroides* spp. has also increased in the past decade; these developments highlight the importance of susceptibility testing in anaerobes [23, 24].

In addition, qualitative and quantitative changes in the human microbiome (including strict anaerobes) have been associated with the development and exacerbation of various chronic diseases, like depression, cardiovascular illnesses, obesity, autoimmune disorders, rheumatoid arthritis, sclerosis multiplex and even autism [25–29]. For this reason, there has been a shift in the interest towards anaerobic bacteria and their virulence factors in the last several decades [19, 30]. The primary requirement for the cultivation, identification and susceptibility-testing of anaerobes is the procurement of an adequate sample from the site of infection, preferably before the onset of antibiotic therapy, and sending the sample to the laboratory for processing as soon as possible [4, 31]. In addition, the availability of appropriate laboratory infrastructure (pre-reduced anaerobically sterilized media for culturing anaerobes, devices capable of generating and maintaining an anaerobic atmospheric such as anaerobic jars and gas generator sachets, Anoxomat™ systems, anaerobic chambers) is essential for the diagnostic procedures of anaerobes [4, 19, 31]. Due to their fastidious growth requirements, the economic considerations required for anaerobic diagnosis and the lack of suitably qualified specialists, species-level identification, antibiotic susceptibility testing and typing of anaerobes were mainly performed in reference laboratories [4]. Nevertheless, the introduction of matrix-assisted laser desorption-ionization time-of-flight mass spectrometry (MALDI-TOF MS) has represented a technological revolution in clinical microbiological diagnostics [32, 33]. This technology allows for protein-based identification of microorganisms, based on the separation and measurement of smaller to larger fragments of highly conserved ribosomal proteins (which are small and basic in character) by their mass to charge *(m/z)* ratio [34]. In the MALDI-TOF MS measurements, the protein spectrum of the clinical isolate is compared with the protein spectrum of strains in the device-linked database and expressed as a log score (microFlex; Bruker Daltonics) or as a percentage (VITEK MS; bioMérieux), which provides information on the level of match and security of identification [35, 36]. Although the introduction of this method was initially largely hindered by the high cost of the device, it is now being used in more and more laboratories worldwide [37]. The MALDI-TOF MS method (along with 16S RNA gene sequencing) has now become the gold standard method of anaerobic diagnostics, providing fast, accurate and reliable results at a low sample cost, and the laboratory can therefore provide information to the physicians within clinically relevant time intervals (compared to presumptive biochemical methods and kits) [38, 39]. Numerous international initiatives have focused on the development and improvement of MALDI-TOF databases on anaerobic pathogens (e.g., ENRIA: European Network for Rapid Identification of Anaerobic Infections), allowing species-level identification of pathogens that were previously not possible [40, 41].

Anaerobic bacteria are important etiological agents in some focal or invasive infections and bacteremia with a high level of mortality; however, conflicting data has accumulated over the last decades regarding the extent in which these pathogens play an intrinsic role in bloodstream infections. The purpose of this review article is to summarize the currently available literature data on the prevalence of anaerobic bacteremia in adults for physicians and clinical microbiologists and to shed some light on the complexity of this topic nowadays.

## ANAEROBIC BACTERIA IN BACTEREMIA

The processing of blood cultures is still considered to be one of the most important tasks of clinical microbiological laboratories [42]. Despite today’s modern healthcare, sepsis, severe sepsis and septic shock still have a high mortality rate (10–15, 20–25, and 40–60%, respectively), during which any delay in the choice of adequate therapy reduces the patient’s chances of survival [43–46]. Blood cultures nowadays are mainly incubated in automated systems, which detect positive results (i.e., generation of bacteria in the sample) by sensing changes in the level of CO_2_ within the bottles. The time from insertion of the bottles to the positive signal is termed time-to-positivity (TTP) of the blood culture, which may be influenced by the generation time of the pathogens, the initial inoculum in the flask, prior antibiotic exposure or the chemical composition of the culture media used in the bottles of the different blood culture systems [47]. The role of consultative microbiology and the continuous professional collaboration between the microbiologist and clinicians is of paramount importance in the treatment of bloodstream infections [48]. Anaerobes play an important etiological role in many invasive infections and may be clinically significant pathogens in bloodstream infections and septicemia, despite their relatively low prevalence [49]. Transient bacteremia with an anaerobic component is frequently associated with tooth extraction and dental surgeries (as the oral microbiome is rich in anaerobic bacteria), but these bacteria are usually eliminated rapidly from the bloodstream by a healthy immune system [50, 51]. It must also be mentioned that the isolation of some anaerobic species from the bloodstream (e.g., *Clostridium septicum*) is significant as it may be the first indicator of colorectal cancer [52].

Clinical characteristics of anaerobic bloodstream infections do not differ from bacteremia caused by other (non-anaerobic) pathogens, but due to the longer generation time and rigorous growth requirements of these bacteria, it usually takes longer to establish the etiological diagnosis [49, 53]. Despite its infrequent occurrence, the mortality rate associated with anaerobic bacteremia still remains very high (ranging between 15 and 50%) [49, 53]. In general, Gram-negative anaerobic rods (predominantly members of the *Bacteroides/Parabacteroides* genus) are the most common in bacteremia, followed by *Clostridium* species; however, virtually the entire spectrum of anaerobic species has been described (at least at a case level) as a pathogen causing clinically significant bacteremia [48, 49, 53, 54]. *Bacteroides/Parabacteroides* bacteremia is generally characterized by thrombophlebitis, the presence of metastatic foci, hyperbilirubinemia, disseminated intravascular coagulation, while *Clostridium* spp. bloodstream infections may include hemoglobinuria, anemia, oliguria, and brownish discoloration of the skin, in addition to the general systemic inflammatory reaction [49, 53]. Numerous reports have reported the effect of inappropriate empiric therapy on the mortality rate of anaerobic sepsis, particularly when the therapy did not include relevant anti-anaerobic agents, or the microorganism was resistant to the empirical antibiotic therapy received [55].

Anaerobic bacteremia mainly affects adults, with elderly patients (>65 years) having high risk for developing bacteremia with this etiology; in contrast, the prevalence of anaerobes in bloodstream infections in neonates and children is extremely rare (0–0.5% overall, with children between 2 and 6 years of age having the least risk) [49, 56]. In general, early studies published between the 1960s and 1980s, the proportion of anaerobes in bacteremia was 20–30%, while between 1980s and 1990s, this value was around 10–20% [49, 57]. However, after the 1990s, the ratio of anaerobic bacteremia has decreased significantly, with literature in the current decade suggesting their proportion to be around 5% on average (ranging between 0.5 and 13%), which corresponds to around 1 episode reported per 1,000 hospitalized patients [49, 58]. This decrease was suggested to have occurred due to the introduction of the prophylactic use of broad-spectrum antibiotic therapy (containing antibiotics with prophylactic anti-anaerobe activity), pre-operative treatments before bowel surgery, and the “predictability” of anaerobic bacteremia, based on the risk factors determined for these infections [48, 49]. Some authors have gone as far as suggesting that anaerobic blood cultures should only be used selectively, if the anamnestic data or clinical signs and symptoms are suggestive of anaerobic bacteremia [59]. In contrast, other studies have suggested that the prevalence of anaerobic bacteremia is actually increasing, corresponding with the higher number of complex and invasive surgical procedures, immunosuppressed patients and patients requiring hospitalization in tertiary-care hospitals [60]. Nevertheless, during the assessment of these epidemiological studies, several variables (geography, differences in the size and composition of study populations, e.g., patient age, social status, underlying immunocompromising conditions; antibiotic policies of the healthcare-institution, treatment level of the hospital, e.g., primary vs. tertiary-care; identification methods used) need to be considered, as these may significantly affect the reported outcomes in these studies [48, 49, 57, 60].

## EPIDEMIOLOGICAL STUDIES ON ANAEROBIC BACTERIA IN ADULTS

In a very early review of 14 studies on anaerobic bloodstream infections published by Finegold *et al.* corresponding to the period between 1956 and 1974 has found, that the female genital tract was the source of anaerobic bacteremia in 20% of cases, while the gastrointestinal tract was the source in almost half of anaerobic bacteremias; sources were unknown in only 6% of cases [61]. Lombardi *et al.* published data regarding the epidemiology University of Michigan Hospitals during 1987–1988; this publication has shown similar findings to the study of Finegold *et al.:* 72% of patients with an anaerobic bloodstream infection had the genito-urinary and gastrointestinal tracts as sources of infection [62]. In addition to these reports, a survey conducted by Morris *et al.* at Duke University Medical Center (a 1125-bed tertiary care hospital) from 1989 to 1991 indicated that the source of infection was clinically obvious in 84% of patients with anaerobic bacteremia [59]. They concluded that because the types of infection causing anaerobic bacteremia were generally predictable, anaerobic blood cultures should only be performed selectively; at the same time other researchers have echoed this recommendation, partly because the rates of anaerobic bloodstream-infections in their studies were rather low. Different members of *Bacteroides/Parabacteroides* spp., especially *Bacteroides fragilis* were the most common blood isolates recovered from patients with anaerobic bacteremia; these organisms accounted for approximately 55% of anaerobic bacteremias. *B. fragilis* bacteremia was associated with intra-abdominal disease and a very high mortality rate (19–40%), a 3.2-fold risk of mortality and prolonged hospital stay. Associated risks for mortality include alcoholism, chronic liver disease and congestive heart failure [59]. Goldstein and Citron determined the relative annual isolation rate of anaerobic bacteria and the susceptibility of *B. fragilis* group species isolated during 1987 at two community hospitals in Los Angeles, California. The relative frequencies of the isolation of *n =* 261 strains were as follows: *B. fragilis* 61.0%; *Bacteroides thetaiotaomicron* 17.0%; *Parabacteroides distasonis* 7.0%; *Bacteroides vulgatus* 6.0%; *Bacteroides ovatus* 5.0%, and *Bacteroides uniformis* 4.0%. They recovered *n* = 8 (18.0%) *Clostridium* spp., and *n* = 2 (4.0%) *Fusobacterium* spp. [63]. One year later, Brook published clinical and microbiologic data about 296 patients with anaerobic bacteremia surveyed over 12 years in two military hospitals in the Greater Washington DC area. Total of *n* = 212 *Bacteroides* spp. were isolated, *B. fragilis* accounted for 78.0%, and *B. thetaiotaomicron* for 14.0% of the cases; among other species, there were 6.0% *Fusobacterium* isolates, 18.0% of various *Clostridium* species and 15.0% of GPAC [64]. The primary source bacteremia in these anaerobic bloodstream infections were the gastrointestinal tract (42.0%), decubitus and gangrene, the female genital tract and the oropharynx (10.0%, respectively). Factors predisposing to anaerobic bacteremia were abscesses and malignancy in case of *n* = 53 patients each, surgery in *n* = 30 patients and intestinal obstruction and/or perforation in a minor group of patients [64]. According to a review of Goldstein in 1996, the data from earlier studies showed that anaerobes account for ~20% of all bacteremia, but newer results showed that these organisms account for approximately 4% (0.5%–9.0%) of bacteremia at that time (or approximately one case per 1,000 admissions) [65]. Salonen *et al.* studied the incidence of anaerobic bloodstream infections over 6 years (1991–1996) retrospectively at the Turku University Central Hospital in Finland. In this report, 4.0% of all bacteremia yielded anaerobic bacteria and the isolation of these microorganisms was clinically significant in 57 patients (0.18 cases per 1,000 admissions) [55]. According to their data, only 50.0% of these patients received effective, appropriate antimicrobial therapy, before the results of blood cultures were reported to the clinicians; 18 patients (32.0%) got initially ineffective treatment, which was later changed, because of the microbiological results and for 11 patients, treatment was not changed, even after microbiological results became available. The mortality in these patient groups were 18.0, 17.0, and 55.0%, respectively [55]. In a study in the United Kingdom between 1969 and 1990, Grandsen *et al.* recovered *n* = 250 anaerobic isolates: 55.0% of these strains were among *B. fragilis* group isolates, 12.0% *Clostridium* spp., 8.0% GPAC, and 7.0% *Fusobacterium* spp [66]. Peraino *et al.* published data of a 350-bed community hospital in Santa Monica, California in 1991. They isolated *n* = 48 different anaerobic strains from 20 patients and found that 6.2% of all positive blood cultures anaerobes in them. 16 patients had clinically significant AB and the outcome was fatal for 44.0% of these patients; two patients died before results could be given to the clinicians [67]. The source of infection was obvious for 68.0% of patients and half of patients were receiving appropriate antimicrobial therapy, active against anaerobes. Their final conclusion was that positive anaerobic blood cultures often resulted in a change in the antimicrobial therapy, even though anaerobic bacteremia was uncommon in their hospital [67]. Between 1987 and 1988, sixty-six patients at the University of Michigan Hospitals (UMH) and nine patients at the Ann Arbor Veteran’s Administration Medical Center (AVMC) in the US were investigated by Lombardi *et al*. [68]. The ratio of positive anaerobe blood cultures was 3.2% at the UMH and 1.8% at AVMC, the incidence of clinically significant AB at the two hospitals were 0.68 and 0.54 per 1,000 patient admissions, respectively. 38.0% of the patients had a fatal outcome; among these, *Bacteroides* and *Clostridium* species accounted for 90.0% of the isolates and in all of the fatal cases. The source for anaerobic bacteremia was usually obvious; gastrointestinal infections were the source in 66.0% of the cases and was clearly implicated as the source of nearly all of the fatal anaerobic bacteremias [68]. Ramos *et al.* reviewed a total of *n* = 231 patients observed over a period of six and a half years in the Fundacion Jimenez Diaz Hospital, Madrid, Spain and *n* = 131 episodes of AB were retrospectively analyzed with special attention given to microbiologic, epidemiologic and clinical risk factors: the frequency of anaerobic bacteremia was relatively high (7.5%) and clinical significance was found in 66.0% of the episodes; attributable mortality with anaerobic bacteremia was 32.0% [69]. The isolation of *Bacteroides/Parabacteroides* spp. was clinically significant in 89.0%, while in case of *Clostridium* spp., this was only 33.0% of cases. The presence of a serious underlying disease or septic shock, renal failure, inappropriate antimicrobial treatment and the absence of drainage or surgical intervention for the septic foci were considered risk factors associated with a bad prognosis [69].

The incidence of anaerobic bacteremia was studied retrospectively, over 62 months (between January 1999 and March 2004) at Mont-Godinne University Hospital, Yvoir (a 380-bed tertiary-care teaching hospital) in Belgium by Blairon *et al*. [70]. During this study, the distribution of organisms, clinical presentations, choice of antimicrobial therapy and clinical outcome were analyzed. The proportion of positive blood cultures yielding obligate anaerobes was 3.3%, the overall incidence of clinically significant anaerobic bacteremia was 0.51 cases/1,000 patient admissions (0.61 cases/10,000 hospital-days); these figures were significantly higher in patients with active hematological malignancies (5.97/10,000 vs. 0.33/10,000 hospital-days). *Bacteroides/Parabacteroides* spp. accounted for 61.0% of isolates, followed by *Clostridium* spp. (12.2%), GPAC and surprisingly the *Leptotrichia* spp. (7.3% each) and *Fusobacterium* spp. (4.8%). In this study, the most common risk-factors were gastrointestinal surgery (half of the patients) and hematological malignancies with chemotherapy and/or bone marrow graft (47.0%), one or more co-morbidities were present in 77.5% of the 39 patients. The lower gastrointestinal tract and the oropharynx were the two most frequent proven sources of bacteremia; the overall mortality rate was 13%. According to their experiences, the fatal outcome correlated with the severity of existent underlying diseases and the immunosuppressed status of the patients, rather than with the causative pathogen or the effectiveness of antimicrobial therapy [70]. Another report from Belgium published by De Keukeleire *et al.* identified the current situation of anaerobic bacteremia in the University Hospital Brussel in a 10-year retrospective study, which presented data between 2004 and 2013. The cases of anaerobic bacteremia per 100,000 patient days decreased from 17.3 in the period from 2004 to 2008 to 13.7 in the period 2009 to 2013, furthermore, the mean incidence of anaerobic bloodstream infections decreased during the study period (1.27/1,000 patients in 2004 vs. 0.94/1,000 patients in 2013) [71]. In case of these two study periods, a total of 437 different anaerobic bacterial strains was isolated, with an average of 33 cases of anaerobic bacteremia per year during 2004–2008, compared to an average of 27 cases per year during 2009–2013 (corresponding to a decrease by 19% between the first and the second study-period). In contrast, the proportion of isolated anaerobic bacteremia, compared to the number of all bacteremia remained stable at 5%. Similarly to previous reports described above, *Bacteroides/Parabacteroides* spp. accounted for 47.1% of the isolates, followed by 14.4% *Clostridium* spp., 12.6% non-spore-forming Gram-positive rods, 10.5% GPAC, 8.2% *Prevotella* spp. and other Gram-negative rods and 7.1% *Fusobacterium* spp. The lower gastrointestinal tract (around 50%) and wound-, skin- and soft-tissue infections were the two most frequent sources for anaerobic bacteremia, while the origin was unknown in 21% of cases; the overall mortality rate was 14% [71]. Kim *et al.* investigated the incidence and risk factors related to mortality and assessed clinical outcomes of anaerobic bacteremia in 2012 on the patients who were hospitalized at Severance Hospital (a 2,000-bed university tertiary referral hospital) in Seoul, Korea. They followed the tendencies in AB retrospectively in this institution since 1974 and found that the incidence of anaerobic blood infections has gradually increased in the last four decades, from 1.39% in 1974–1983 to 1.96% in 2007–2008. As they remarked, there were some important technological changes in blood culture equipment in their institution, blood culture systems from conventional anaerobic broth blood systems to BACTEC 9240 systems (Becton Dickinson Diagnostic Instrument Systems) in 1997, while to BacT/Alert 3D systems (bioMérieux) in 2005, however, the steady increase was observed during these decades [72]. According to their nationwide annual report, the number of elderly patients and patients with serious underlying diseases such as malignancies has steadily increased and indeed, patients admitted to their tertiary care referral center hospital often present with more comorbidities and several advanced diseases. A novel finding in their study was that cardiovascular disease emerged as an important underlying condition, which was significantly associated with higher mortality: the authors assumed that this change in patient population attributed to the increasing trend what was noted [72]. In a publication by Cockerill *et al.*, corresponding to the period between 1984 and 1992, a steady increase in the incidence of anaerobic bacteremia was noted at the Mayo Clinic, and later, another retrospective study report from the same institution also observed an increase in incidence during the subsequent 12-year period (from 1993 through 2004) [73]. In a 12-year study at an Australian general hospital, Riley and Arvavena found a 200% increase in the incidence of anaerobic bacteremia, with *Fusobacterium* species and GPAC being more frequently identified [74].

In contrast, other reports provided no evidence of an increase in the incidence of anaerobic sepsis or bacteremia: Chandler *et al.* reviewed the relevance of blood cultures in a 5-year (1994–1999) retrospective study, in context of an older population: they found that the prevalence of anaerobic bacteria may be as low as 0.14%, and in 92% of cases, the anaerobic infection could be suspected based on clinical presentation of the patients [75]. Similar results were shown by Ortiz *et al.* in a 3-year study (1994–1996), where the prevalence of anaerobic bacteremia was lower than 0.5% and all these cases had an obvious source of infection [76]. To highlight the minor role of anaerobic bacteremia in children, Gené *et al.* showed that anaerobes only represented 0.02% of isolates from anaerobically incubated blood cultures over a 2-year period [77]. Fenner *et al.* retrospectively analyzed blood culture data for a 10-year period between 1997 and 2006 from University Hospital Basel, Switzerland (a 680-bed tertiary care center in Switzerland, with 27,000 inpatients and 167,000 outpatients per year). A total of 114,338 blood cultures were submitted to the laboratory, from which, 1,084 (0.95%) anaerobic organisms were isolated; they have found that the number of positive anaerobic blood cultures decreased in the period from 1997 to 2001 (12.6 per 1,000 blood cultures performed) to 7.0, compared to the period from 2002 to 2006. Similar observations were found, if the proportion of isolated anaerobic organisms were compared to the number of all organisms isolated from blood cultures (7.6%–4.3%). The number of patients with anaerobic bacteremia significantly decreased from *n =* 122 in 1997 to *n =* 69 in 2006, but on the other hand, the proportion of *Bacteroides/Parabacteroides* spp. and GPAC increased (26.8%–36.7% and 5.4%–12% respectively) [78]. Authors from the St. Barnabas Hospital, a 450-bed community hospital in the Bronx, New York, which served a predominantly black and Hispanic community, reviewed their data with anaerobic bacteremia during 2000–2006. This health institution provided care for patients in various medical fields (internal medicine, surgery, oncology, substance abuse, psychiatric, obstetrics and gynecology, neonatal medicine), but the oncology service accounts only for <1% of admissions [79]. The authors did not find an increase in the incidence of anaerobic bacteremia in the study period: anaerobic organisms accounted for less, than 2% of positive blood culture results (range: 0.7–1.3%) and the number of positive anaerobic culture results per 1,000 blood cultures performed was 0.73, which is less than the rate of 1.68 positive results per 1,000 blood cultures that was reported by Lassmann *et al.* for the period between 1993 and 1996 [60]. *B. fragilis* accounted for 33.0% of anaerobes, followed by GPAC (19.0%). The etiology of infections was unknown in 42.0% of the cases, 32.0% of cases had an abdominal or urogenital source, whereas 23.5% of cases involved skin and soft-tissue infections. The anaerobic blood culture bottle is routinely used in Japan with little discussion as to its justification or validity. Saito *et al.* retrospectively studied the incidence of anaerobic bloodstream infections and the potential risk factors of AB during a 2-year period (1999–2000) at four university hospitals and one community hospital in Japan. Thirty-four of 18,310 aerobic and anaerobic blood culture sets from 6,215 patients taken at the university hospitals, and 35 of 2,464 samples taken from 838 patients at the community hospital, yielded obligate anaerobes. *Bacteroides* species and *Clostridium* species accounted for 60% of the isolates. Fifty-seven patients from 69 blood culture sets containing anaerobes had clinically significant anaerobic bacteremia, among these 57 patients, almost half were oncology patients, 40 (70%) had an obvious source of anaerobic infection, 15 (26%) had recent surgery and/or were in an immunosuppressed state. Their recovery rate of isolated obligate anaerobes was low and the patients with anaerobic bacteremia had limited number of underlying diseases or potential risk factors for anaerobic infections [80]. Another Japanese study made by Iwata *et al.* performed a retrospective chart review at a private hospital for patients admitted between July 1, 2004 and June 30, 2005 to determine patient characteristics resulting in anaerobic blood culture. During the study period, 17,775 blood culture bottles were sent for analysis, and 2,132 bottles (12.0%) were positive for microbial growth. Only 47 cases were detected by anaerobic cultures alone, among those, obligate anaerobes represented 12 cases [81]. Clinical evaluation could have predicted 7 of 12 cases of anaerobic bacteremia, in the remaining 5 cases, the source of bacteremia was unclear. There were 2.7 cases of anaerobic bacteremia per 1,000 blood cultures. The mortality attributable to anaerobic bacteremia was very high (50%). As a conclusion, all the abovementioned studies have suggested that the selective use of anaerobic blood cultures needs to be considered, instead of the traditionally used setup of taking blood culture samples for both aerobic and anaerobic workup. According to their suggestions, the patients could be identified clinically; and if it is likely that they will have anaerobic bacteremia with a high degree of predictability, they should be treated empirically without the need for microbiological confirmation [75–81].

Nevertheless, several reports highlight that anaerobic bacteremia may often be missed on the basis of clinical findings, which result in patients receiving inadequate antimicrobial treatment [48, 49, 82]. This is especially true for patients undergoing invasive surgical interventions (resulting in the disruption of physical barriers) and intensive cytotoxic chemotherapy regimens (causing profound neutropenia); in these cases, the normal microflora of the patients may enter into the bloodstream, causing bacteremia [48, 49, 82]. In the clinical study of Zahar *et al.* only one-third of patients received appropriate antibiotic therapy before the availability of microbiology results, and around the same amount of patients never received appropriate therapy. *B. fragilis* was represented as the most common isolate, and the overall mortality rate in this study was high (42%) [83]. Minces *et al.* collected cases of bacteremia and endocarditis caused by *Peptostreptococcus* spp. and found that most of affected patients suffered from some type of malignancy [84]. Umemura *et al.* compared the clinical characteristics of patients with anaerobic bacteremia with those with aerobic bacteremia between January 1999 to December 2012 in Aichi Medical University Hospital, Japan. Clinical information for 71 patients corresponding to anaerobic bacteremia was collected and they found an association between anaerobic bacteremia and malignancy, Douglas’ pouch drains and chest drains as the primary causative of bacteremia, as well as associations between anaerobic bacteremia and the gastrointestinal tract; however, having a central venous catheter was not associated with anaerobic bacteremia [85]. In the same institution between January 2005 and December 2014 they treated 74 patients with anaerobic bacteremia. This later retrospective case-controlled study they performed to assess the prognostic factors associated with death from anaerobic bacteremia, the clinical and microbiological information included antibiotic susceptibility was used for analysis of prognostic factors for a 30-day mortality. They found the association between the 30-day mortality rate and malignancy and clindamycin resistance [86]. Anaerobic bacteremia was studied in *n =* 32 patients in a four-year retrospective analysis by Kornowski *et al.* between 1984 and 1988 among internal medicine patients in a 700-bed University Hospital, Aviv, Israel. Overall, anaerobic bacteria accounted for 4.5% of positive blood cultures from the medicine service during this period, the main causative organisms were among the *Clostridium* and *Bacteroides/Parabacteroides* spp. AB occurred either following invasive (non-surgical) procedures or spontaneously; the gastrointestinal tract was affected most often, followed by the respiratory and urinary tracts and malignancy was the most common underlying disease. The fatality rate was 25.0% (but their patients’ mean age was 72 years) [87]. An additional study from Israel by Lazarovitch *et al.* from the Harofeh University Medical Center, Zeriffin, investigated the prevalence of anaerobic bacteremias and evaluated the importance of anaerobic blood cultures from January 1998 to December 2007. AB and sepsis decreased during that period, but significant increase was observed the proportion of *Bacteroides/Parabacteroides* species isolated from blood cultures (from 18.0% during 1998–2002 to 43.0% during 2003–2007). Comparison of the medical data of *n =* 54 patients with *Bacteroides*-related bacteremia during the two study periods (1998–1999 and 2006–2007) revealed a marked increase in serious and heterogenous underlying diseases. Hypertension and Type II diabetes were found in 29.0% of the patients in 1998–1999 and increased to 43–45% of the patients in 2006–2007, while ischemic heart disease also increased from 14.0% in 1998–1999 to 43.0% in 2006–2007. Their conclusion was that, despite the fact that positive anaerobic blood cultures account for a small amount of all positive blood cultures, the growing involvement of *Bacteroides/Parabacteroides* species-related bacteremias together with an increased ratio of complex underlying diseases in these patients emphasize the importance of anaerobic blood cultures [88]. Arzese *et al.* found *n =* 225 anaerobic isolates in a nationwide survey of anaerobic bacteremia in Italy, between 1991 and 1992: 34.0% of anaerobic isolates were members of the *Bacteroides/Parabacteroides* spp., 11.0% *Clostridium* spp., 8.0% GPAC and 6.0% *Fusobacterium* spp. In the other subsequent Italian study from a Northern-Bari Hospital, which was conducted between 2008 and the first quarter of 2013, twenty-six patients were found positive for anaerobic bacteria with an average of 1.28% of positive anaerobic blood cultures [89]. Their analysis shows that the percentage of blood cultures positive for anaerobes was constant temporally, except for a small drop in 2012, despite the greater number of blood cultures being tested. Most cases of sepsis were caused by anaerobic bacteria belonging to the *Bacteroides/Parabacteroides* spp.; however, they found a high incidence of events caused by *C. acnes* [89]. Grohs *et al.* also aimed to ascertain the relevance of routine anaerobic blood cultures in France: during 2004, peripheral blood samples were incubated in a BacT/Alert system. In their report, 13.7% of patients had a positive blood culture overall, including 1.2% strict anaerobic bacteria. In addition to pointing out that anaerobes are important etiological agents, the relevance of anaerobic blood cultures were further shown in their institution, as facultative anaerobes had shorted TTP values in these bottles [90]. Vena *et al.* aimed to perform a retrospective analysis of 10-years’ experience in a tertiary University hospital in Madrid, Spain to investigate the incidence, prognosis and need to perform blood cultures for anaerobic bacteria from 2003 to 2012. Overall incidence of anaerobic bacteremia was 1.2 episodes/1,000 admissions, with no significant changes during the 10-year study period; similarly to findings of other studies, *B. fragilis* group (38.1%) and *Clostridium* spp. (13.7%) were the most frequent isolated microorganisms. 43.4% of the patients had a comorbidity classified as ultimately fatal or rapidly fatal, clinical manifestations suggestive of anaerobic involvement were present in only 55.0% of the patients, however, 24.8% of their patients died during the hospitalization. Independent predictive factors of mortality were presentation with septic shock, whereas, an adequate source control of the infection was associated with a better outcome [56]. Anuradha *et al.* reviewed cases of anaerobic bacteremia over a two-year period in Mumbai, India. Out of *n =* 93 blood cultures received with a suspicion of anaerobic bacteremia, only 18.3% showed anaerobic growth. *n =* 20 anaerobes were detected as single isolates, while five had a polymicrobial flora; the anaerobes isolated were GPAC, *B. fragilis* group, *Bilophila*, and *Eubacterium* species. Seven of these patients (4.3%) had a pre-existing heart condition, while others had a prior history of surgery, diabetes mellitus or urinary tract infection; the oropharynx was the most frequent portal of entry, followed by the gastrointestinal tract. In this study, fifteen patients developed major complications, such as congestive cardiac failure, systemic embolization and perforative peritonitis, the mortality rate among the cases of anaerobic bacteremia was 23.5% [91]. Muttaiyah *et al.* investigated a 2-year study period at the Auckland City Hospital, Auckland, New Zealand: anaerobes were isolated from *n =* 140 blood culture sets, taken from 114 patients, in *n =* 59, these isolates were considered as contaminants; of their note, all *Cutibacterium* spp. were considered as contaminants. In patients with true anaerobic bacteremia, the most likely source of infection was intra-abdominal (50.0%), mucositis associated with neutropenia, contributed to by cytotoxic therapy (19.0%), and a smaller number of cases associated with skin and soft tissue-, pelvic- and oropharyngeal infections. Thirty-five patients were on appropriate therapy, prior to the availability of culture results, *n =* 5 patients died, but only one death was directly attributable to AB; antimicrobial therapy provided appropriate cover for two-thirds of the patients [92]. In Hungary, two studies are available from the same institution in two distinct time periods; in the report by Urbán *et al., n =* 305 anaerobic species were isolated, corresponding to the period between 2005 and 2009, which a pronounced decreasing tendency in the ratio of anaerobic isolates during the 5-year period (from 6.3% to 4.0%). Among the clinically-relevant isolates, *Clostridium* spp. and *Bacteroides/Parabacteroides* spp. were the most common, however, the majority (57.7%) of isolates were *Cutibacterium* spp., reported as contaminants. The average age of affected patients were 60 years and the crude 30-day mortality rate was shown to be 22.3% [48]. Gajdács *et al.* performed a similar epidemiological study, corresponding to the time period between 2013 and 2017; in this 5-year period, *n =* 423 strict anaerobes isolated, corresponding to 3.3–3.6% of positive blood culture isolates or 1 per 1,000 hospitalized patients [93]. In the second study period, the average age of the patients increased significantly (72 years), while the species-distribution did not change drastically, compared to the study of Urbán *et al.* [48, 93]. However, it must be noted that novel anaerobic species, not detected in blood cultures previously in this geographical region, were reported in this study, owing to the introduction of a MALDI-based diagnostic platform [93]. Some studies aimed to assess the epidemiology of specific anaerobic pathogens: in a study from Sweden, Badri *et al.* retrospectively assessed the clinical and microbiological features of anaerobic bacteremia in adults, caused by GPACs between 2012 and 2016; *n =* 226 episodes of GPAC bacteremia (3.4 cases were recorded by using MALDI-TOF MS and 16S rRNA gene sequencing as diagnostic modalities. In this study, the 30-day crude mortality was 11%, while the most common species were *Anaerococcus* spp. (>50%) [94]. In a hospital-based case series, Almohaya *et al.* highlighted the increase in the numbers of Lemierre syndrome and bacteremia caused by *Fusobacterium* spp. in Saudi Arabia; during their analysis, the authors found 205 individual cases reported from their country, in addition on the two cases they have presented between 2015 and 2019 [95]. A study by Stabler *et al.* from France evaluated the relevance of *Clostridium* spp. bacteremia between 2010 and 2018, including *n =* 81 patients, with at least one positive anaerobic blood culture for *Clostridium* spp.; the 30-day crude mortality observed in patients was 31.4%, and the administration of the adequate antibiotic therapy was associated with increased survival *(P =* 0.03) [96]. Finally, a recently published article from France by Lafaurie *et al.* noted *n =* 209 positive anaerobic bottles in a 6-month survey period: most of the isolates (60.3%) were contaminants, while true anaerobic bacteremia was detected in 13 patients (out of which 9 had underlying gastro-intestinal illness) [97].

## ROLE OF ADVANCES IN DIAGNOSTIC PROCEDURES

Previously, the identification of strict anaerobes in clinical samples mainly relied on in-house, classical biochemical testing, biochemical test strips (e.g., API ID32A Kit, RapidID ANA II System) or automated systems (VITEK ANC Card) and gas-liquid chromatography (GLC) [19, 98, 99]. These methods were quite pricy, were available in only a few diagnostic laboratories and provided identification results only 48–72 hours later, mostly on the genus level [19, 98, 99]. Recent publications have pointed out that the introduction of novel technological modalities (e.g., PCR, MALDI-TOF MS, 16S rRNA sequencing, automation) into the routine diagnostic workflow affected both the qualitative and quantitative aspects of anaerobic bacteremia [19, 100–102]. As these technologies have become available to a growing number of laboratories, rapid, accurate and reliable identification of anaerobes on species-level in a clinically-relevant time-frame has become more common [32–36]. MALDI-based analysis may also have application in detection of resistance in anaerobes (e.g., differentiation of *cfiA-*negative and cfiA-positive *B. fragilis* strains [103]) or in typing (e.g., discrimination of different phylotypes of *C. acnes* [104]). Shannon *et al.* have also reported on the methodology called “early MALDI”, where short-term (4–6 hours) incubation of subcultures of positive anaerobic blood culture bottles was shown to be a reliable method for at least genus-level identification of common anaerobic species. The study group has concluded that the utilization and perfection of the “early MALDI” highlights the role of mass spectrometric analysis as a superior and more cost-effective method for identification than sequencing [105]. In addition, there have been continuous developments in improving and complementing databases of bacterial spectra for MALDI-TOF analysis, to also be appropriate for the detection and differentiation of rarely occurring or taxonomically close microorganisms [106]. In parallel, the characterization of yet unknown bacterial species in the microbiome of humans has occurred with the use of metagenomic technologies and next-generation sequencing; since the 2010s, as many as 200–300 novel bacterial species *(spec. nov.)* are being annotated each year [107, 108]. As a result of these developments, several “new”, so far unknown anaerobic species have been described as causative agents in bacteremia and invasive infections, which were not previously reported as possible pathogens, resulting in an explosion of publications and case reports. As an example, in our most recent study between 2013 and 2017, five different species of Gram-positive anaerobes (namely *Actinotignum schaali, Collinsella aerofaciens, Flavonifractor plautii, Solobacterium moorei* and *Tissierella praeacuta)* were described as causative agents in bacteremia, which had not been previously reported in Hungary before; in addition, compared to the previous study performed between 2005 and 2009, a significantly higher number of species was detected in the more recent report (26 vs. 38 different species) [48, 93]. Nonetheless, the publishing of the “first reports” of clinical relevance in bacteremia for *A. schaali* [109], *Anaerobiospirillum succiniproducens* [110], *Butyricimonas virosa* [111], *Capnocytophaga gingivalis* [112], *Desulfovibrio desulfuricans* [113], *Dysgonomonas mossii* [114], *Fenollaria massiliensis* [115], *Propionimicrobium lymphophilum* [116], *S. moorei* [117], *Slackia exigua* [118] and *T. praeacuta* [119] among others, were possible due to mass spectrometry-based bacteriological diagnostics. It should be expected that further novel “emerging” strict anaerobic species will be described as pathogens in bacteremia in the coming years.

## CONCLUSIONS

Anaerobes are important components of the conventional human microbiota and they are also common etiological agents in the infections of virtually all anatomical sites, including bacteremia. The early recognition and adequate therapy of these infections is of great importance, as the mortality rate associated with these infections is still pronounced. The aim of this present review was to summarize the literature available on the epidemiology of anaerobic bacteremia in adults. As presented above, conflicting data have accumulated in the literature regarding the incidence of anaerobic bacteremia and on the relevance of the routine use of anaerobic blood cultures. The latter may be helpful when obligate anaerobes give rise to bacteremia which may be clinically suspected in patients with advanced age and/or in severely immunocompromised states, having undergone complex surgeries or having serious underlying diseases. The introduction of MALDI-TOF MS and sequencing has changed the face of diagnostic microbiology, which has a definite effect on anaerobic bacteriology. In addition, cultivation of these bacteria is also important for susceptibility-testing purposes, as many anaerobic species besides the *Bacteroides/Parabacteroides* spp. have developed beta-lactamase activity; with multiple changes in the resistance patterns of anaerobes, one can expect that therapeutic problems in the future will be compounded by abandonment of the “complete bacteriology” of blood cultures. The prevalence of anaerobic bacteremia in relation to patient demographics should be determined on an institution by an institutional basis to guide blood-culture practices. This approach will ensure correct diagnosis and that patients will receive appropriate therapy.
